# Burden of functional difficulties among children aged 2–17 years in Somalia: analysis of a 2022 nationally representative survey data

**DOI:** 10.3389/fpubh.2026.1871528

**Published:** 2026-07-30

**Authors:** Hassan Abdi Ahmed, Nelson Jerry Ndifwa, Abdimalik Ali Warsame, Jama Barre Hared

**Affiliations:** 1 Center for Graduate Studies, Department of Statistics and Data Analytics, Jamhuriya University of Science & Technology, Mogadishu, Somalia; 2 Eastern Africa Statistical Training Centre, Dar es Salaam, Tanzania; 3 Faculty of Economics and Management Sciences, Hormuud University, Mogadishu, Somalia

**Keywords:** children, determinants, functional difficulty, prevalence, Somalia

## Abstract

**Background:**

Functional difficulty is a substantial public health concern. Empirical data on children experiencing difficulty are indispensable for developing or monitoring laws, policies, strategies, and interventions intended to mitigate disability; however, evidence on the magnitude and underlying determinants affecting children in Somalia remains limited.

**Objective:**

This study aimed to assess the prevalence and contributing determinants of functional difficulties among children aged 2–17 years in Somalia.

**Methods:**

A cross-sectional analysis was conducted using data from the 2022 Somali Integrated Household Budget Survey (SIHBS). A multiple logit model was run, reporting adjusted odds ratios (AOR) with 95% confidence intervals (CIs) at *p* < 0.05.

**Results:**

The overall prevalence of functional difficulty among children was 5.7% [95% CI: 5.4–6.0]. Household size (AOR = 0.62, 95% CI [0.44, 0.89]), illness status (AOR = 0.46, 95% CI [0.35, 0.60]), and school attendance (AOR = 1.59, 95% CI [1.21, 2.09]). Chronic disease (AOR = 0.12, 95% CI [0.08, 0.19]), poverty status (AOR = 0.62, 95% CI [0.48, 0.81]), and residence (AOR = 1.18, 95% CI [0.99, 1.41]) were all significantly associated with functional difficulty at *p* < 0.05.

**Conclusion:**

Functional difficulties among Somali children are shaped by health, socioeconomic, and residential factors. Strengthening disability policies, expanding screening and mobile health services, promoting inclusive education, and providing targeted social protection are essential to address this burden.

## Introduction

Functional difficulties in childhood can have profound effects on children’s physical health, psychosocial well-being, educational participation, and overall development, with broader implications for families and communities. Disability is understood as a multidimensional concept encompassing impairments, activity limitations, and participation restrictions that result from the interaction between individual characteristics and environmental barriers (1, 2). In line with this perspective, the United Nations Sustainable Development Goals emphasize the importance of inclusive, equitable, and quality education and lifelong learning opportunities for all children and adolescents, including those with disabilities (3). Despite these commitments, children and adults with disabilities continue to experience poorer health outcomes, greater social exclusion, stigma, and reduced opportunities for education and economic participation, which may contribute to reduced quality of life and life expectancy (4). The UNICEF/Washington Group Child Functioning Module (CFM) provides a standardized approach for assessing child functioning and disability by identifying difficulties in key domains, including vision, hearing, mobility, communication, memory, and concentration, that may limit children’s ability to perform essential daily activities (5, 6).

The World Health Organization (WHO) estimates that nearly one billion people globally, accounting for approximately 15% of the world’s population, experience some form of disability, with the highest concentration occurring in low- and middle-income settings where access to supportive services and resources is often constrained (5, 7). Among children, approximately 240 million worldwide are estimated to live with functional difficulties, with the greatest prevalence observed in the West African region (8). Advancing the 2030 Agenda’s commitment to ensuring that no individual is excluded from development efforts requires a comprehensive understanding of the health conditions, service needs, and care-seeking patterns of children with disabilities, particularly in vulnerable contexts (9). However, accurately quantifying the global and regional burden of childhood disability remains challenging due to differences in measurement methods, limited availability of robust population-based data, and inadequate integration of disability indicators into routine data systems. Expanding disability-inclusive data collection and strengthening the use of standardized assessment tools are therefore critical for generating reliable evidence, informing policy decisions, and designing responsive interventions (8). Worldwide estimates from UNICEF demonstrate considerable regional variation in the number of children aged 0–17 years living with disabilities, including 10.8 million in Europe and Central Asia, 8.0 million in North America, 19.1 million in Latin America and the Caribbean, 43.1 million in East Asia and the Pacific, 64.4 million in South Asia, 20.9 million in the Middle East and North Africa, 41.1 million in West and Central Africa, and 28.9 million in Eastern and Southern Africa (8). These variations underscore the importance of generating locally relevant evidence to identify population-specific risks, address structural barriers, and promote inclusive health, education, and social protection systems for children with functional difficulties.

Globally, over 290 million children and adolescents experience functional difficulties, meaning that more than one in ten have conditions such as cognitive limitations, epilepsy, or hearing and vision loss. Moreover, the vast majority, 94.5% (approximately 275.2 million) of these children live in low- and middle-income countries (10). A vast number of children with disabilities reside in low- and middle-income countries and face lower school enrollment rates; those attended school are more prone to dropping out before finishing primary or secondary education, creating significant obstacles to employment and economic participation (10). In 2017, approximately 291 million children and adolescents worldwide (11.2%) were living with one of four disabilities. Prevalence increased with age, rising from 6.1% among infants to 13.9% among adolescents aged 15–19 years. Most affected children (94.5%) lived in low- and middle-income countries, primarily in South Asia and sub-Saharan Africa, with ten countries accounting for 62.3% of cases (10).

In 2016, it was estimated that approximately 8% of children worldwide experienced developmental difficulties (11). These difficulties involve conditions including hearing and vision loss, epilepsy, cerebral palsy, autism, intellectual disability, and other learning disorders, which affect children’s physical, learning, and behavioral abilities (12). Children with these conditions are more likely to experience functional difficulties, such as impairments in vision, hearing, mobility, concentration, cognition, behaviour, communication, memory, and self-care (13). Based on the existing literature, the study considered the following sociodemographic variables: sex (12), age (14), household size (15), school attendance (15), having an illness (16), and chronic disease status (17), as well as poverty status and geographic factors, including type of residence and region (1).

In Somalia, people with disabilities experience marginalization across all aspects of humanitarian response, including economic, social, and cultural rights. They are among the most vulnerable members of society, facing social stigma, inaccessibility, discrimination, and exclusion. As a result, they often have poorer health outcomes, lower educational attainment, and limited economic opportunities compared to their physically able peers, largely due to restricted access to essential services. Families of persons with disabilities also encounter challenges stemming from stigma and the lack of supportive services and opportunities for their family members (18). Many children with disabilities are excluded from formal education and remain confined at home, often hidden from public view. Both children and adults with disabilities are subject to social stigma, segregation, and derogatory labeling. Ongoing conflict further exacerbates these barriers, with girls and internally displaced persons (IDPs) with disabilities being disproportionately affected (18). To boost the socio-economic and environmental well-being of people with disabilities, the Somali government has established legal frameworks and policies and has implemented or supervised social protection and development programs. The Provisional Constitution of the Federal Government (Republic) of Somalia (FGS) guarantees equal legal rights for persons with disabilities and prohibits the state from engaging in discrimination against them (19).

Prior studies on disability remain very limited in Somalia, largely due to the scarcity of reliable and comprehensive data. Only a small number of studies have been conducted on it, and the existing literature is often outdated, providing an incomplete understanding of the issue. Furthermore, these studies have largely overlooked the functional difficulties faced by children with disabilities across the country, leaving significant gaps in knowledge regarding their prevalence, determinants, and the challenges they encounter in daily life. This lack of up-to-date and nationwide evidence hampers efforts to develop targeted policies and interventions to support children with disabilities effectively. To our knowledge, limited research has examined factors associated with functional difficulties among children aged 2–17 years in Somalia, whereas existing studies on disability are typically localized, limited to specific regions, and often lack the use of advanced statistical and spatial methods needed to capture the complexity of the issue across the country’s diverse contexts. Therefore, this study aimed to estimate the national burden of functional difficulties among children and identify the associated determinants using a nationally representative sample from Somalia. Estimates from this study are crucial for guiding effective policy development and the implementation of targeted interventions.

## Methods

### Data, design and sample size

A cross-sectional study design was employed to estimate the burden of functional difficulties and identify associated determinants, using secondary data from the 2022 Somali Household Budget Survey, a nationally representative dataset (20). The target population comprised all children residing in Somalia, while the study population included children aged 2–17 years identified through the 2022 Somali Household Budget Survey. The final analytical sample consisted of 25,078 children from 601 enumeration areas across 17 regions, providing national and regional representation. A stratified multi-stage sampling approach was implemented to capture urban, rural, and nomadic populations (21). In urban and rural settings, a three-stage cluster sampling procedure was used, with primary sampling units selected using probability proportional to size, followed by household listing and systematic household selection. In nomadic areas, a two-stage stratified sampling design was applied, whereby 12 households were randomly selected from each chosen enumeration area (22, 23).

## Measurement of functional difficulty

### Outcome

**Figure 1:**
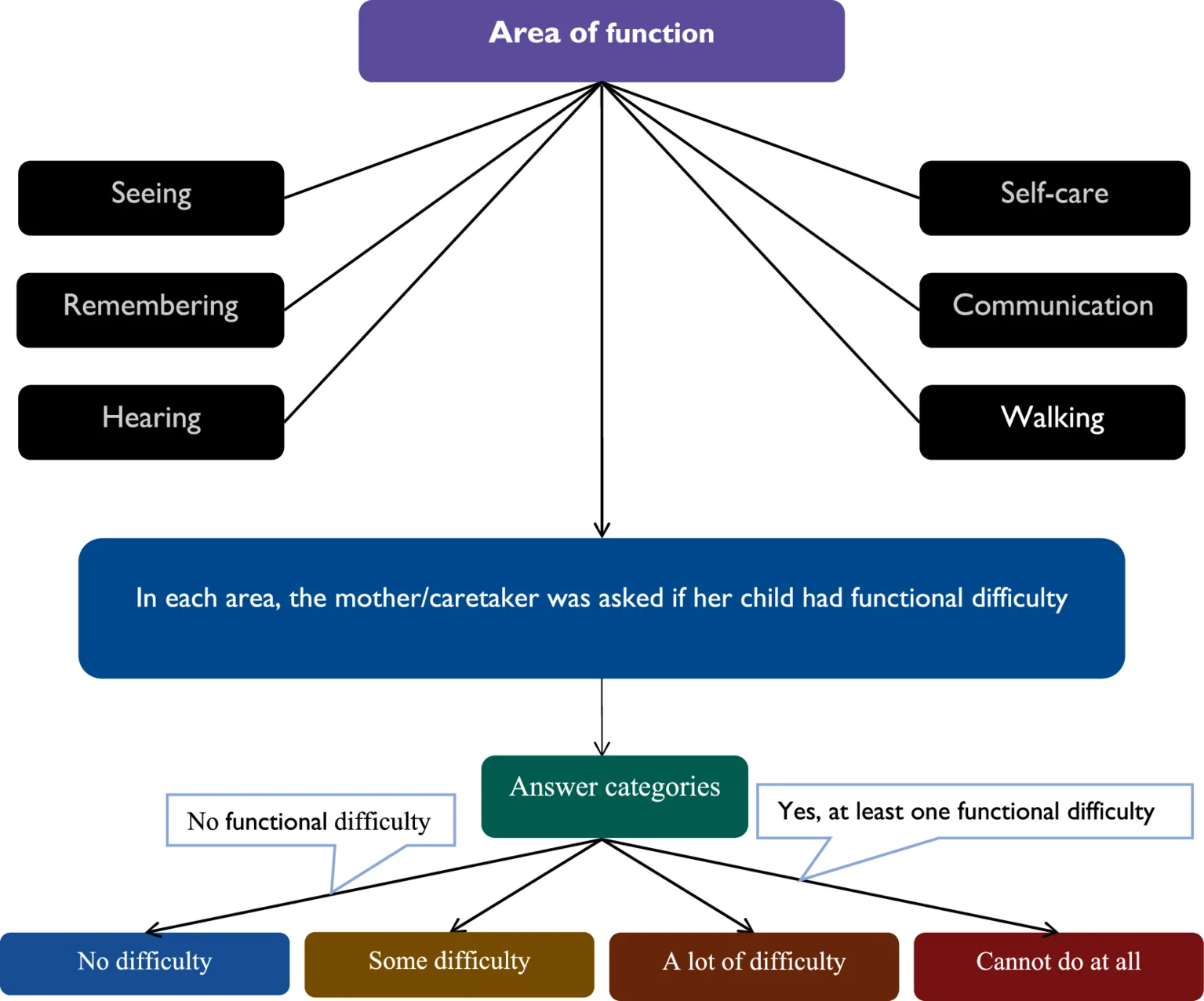
The conceptual framework for child functional difficulty across six functional domains.

Child functional difficulty was measured using the Washington Group (WG) Child Functioning Module (CFM), a standardized and internationally validated instrument developed to identify functional difficulties and disabilities among children across multiple domains of functioning (6, 24). The CFM is widely used in national surveys and international monitoring frameworks to generate comparable data on child disability and functional limitations across diverse populations (24). The WGCFM evaluates functional difficulties across domains, including vision, hearing, mobility, communication, learning, remembering, concentrating, self-care, behaviour, relationships, and emotions, depending on the child’s age group (25). Responses are recorded using standardized categories that reflect the degree of difficulty experienced (6, 26). For the present study, the outcome variable was derived from six functional domains available in the 2022 Somali Integrated Household Budget Survey (SIHBS): difficulty seeing, difficulty hearing, difficulty walking or climbing steps, difficulty remembering or concentrating, difficulty with self-care, and difficulty communicating (20). Children reported as having “no difficulty” across all six domains were classified as not having a functional difficulty (coded as 0 = No). Conversely, children reported as having “some difficulty,” “a lot of difficulty,” or being “unable to do at all” in at least one domain were classified as having a functional difficulty (coded as 1 = Yes). Consistent with previous studies and the Washington Group recommendations, the outcome variable was operationalized as a binary measure for regression analysis (0 = No functional difficulty; 1 = At least one functional difficulty) (1, 6, 25, 27). Responses recorded as “do not know” were excluded from the analysis to minimize potential misclassification and enhance the validity of the findings. The conceptual framework and classification of child functional difficulties across the six domains are presented in Figure 1 (20). This study focused on six functional domains derived from the SIHBS, which may not fully capture all aspects of child functioning assessed in the full Washington Group Child Functioning Module. As a result, the burden of functional difficulties may be underestimated. All variables included in the analysis for children aged 2–17 years had complete observations; therefore, no missing data imputation or handling procedures were necessary. The conceptual framework for child functional difficulty is presented in Figure 1.

### Covariates

Drawing from prior research literature, the following nine explanatory variables were addressed in this study (1, 15, 21) (Table 1). For poverty measurement, three key components were required. First, an appropriate measure of household well-being was selected, typically based on income, consumption, or an asset-based index, depending on data availability and context. Second, a poverty threshold (or cutoff point) was established to distinguish households classified as poor from those considered non-poor. Third, a composite poverty indicator was constructed to summarize overall poverty status by combining relevant dimensions of deprivation into a single measure for analytical purposes (21, 28). In this study, poverty status was derived from the SIHBS 2022 and operationalized as a dichotomous variable (0 = non-poor households and 1 = poor households) (20). At the outset, place of residence was classified as urban, rural and nomadic; however, the nomadic category was merged with rural due to the small number of observations to ensure adequacy and stability of estimates in the regression analysis. The variable was therefore re-categorized as urban and rural (21).

**Table 1:** Study variables, definitions, and descriptions used in the analysis of child functional difficulties in Somalia.

Covariate	Description
Sex	Sex of the child (1 = male, 2 = female) (27).
Age	Age of the child (1 = 2–4 years, 2 = 5–9 years, 3 = 10–14 years, 4 = 15–17 years (1, 6, 48)
Household size	Household size (1 = 1–3 members, 2 = 4–6 members, 3 = ≥7 members) (26, 49).
Suffering illness	The child’s recent illness (1 = Yes, 2 = No) (16)
School attendance	School attendance of the child (1 = Yes, 2 = No) (50)
Chronic illness status	Chronic illness status of the child (1 = Yes, 2 = No) (17)
Poverty status	Poverty status of the household (0 = Not poor, 1 = Poor) (34).
Type of residence	Type of residence of the child (1 = Urban, 2 = Rural) (1).
Region	Region (1 = Northern, 2 = Central, 3 = Southern) (51).

### Inclusion and exclusion criteria

This study applied the Washington Group (WG) Child Functioning Module (CFM) framework to measure child functioning and disability, which is designed to enhance the quality, consistency, and international comparability of disability measurement in censuses and national surveys (24). The WG framework provides standardized indicators for capturing functional limitations across populations. Children younger than 2 years of age were excluded from the analysis due to the high likelihood of misclassification associated with normal developmental variation at early ages (24). Consistent with WG recommendations, the child functioning module targets children aged 2–17 years (1). Accordingly, this study was restricted to children within this age range (2–17 years), as extracted from the Somali Integrated Household Budget Survey data conducted by the Somalia National Bureau of Statistics (20).

### Statistical analysis

Children aged 2–17 years were employed as the unit of analysis. First, descriptive statistics were generated, such as weighted frequencies and percentages, to account for the survey design. Bivariate logit regression was then performed to examine associations between the outcome (functional difficulty) and explanatory factors. Multicollinearity was assessed using the Variance Inflation Factor (VIF), and no evidence of multicollinearity was detected, as all VIF values were below the threshold of 5. Variables with a *p*-value less than 0.25 in the bivariate analysis were subsequently included in the multiple logistic regression model, and all met the inclusion criteria. In the multiple analysis, adjusted odds ratio (AOR) with 95% confidence intervals (CIs) were estimated, and statistical significance was determined at *p* < 0.05. All analyses were done using Stata version 17.0 (StataCorp LLC, College Station, TX, USA), while Microsoft Excel was used for visualization.

## Results

### Prevalence of functional difficulties among children aged 2–17 years in Somalia

Table 2 presents that 94.33% [0.940, 0.946] of children aged 2–17 years had no functional difficulty, whereas 5.7% [0.054, 0.060] of children experienced at least one functional difficulty. Although the overall prevalence is relatively low, this still represents a meaningful proportion of children who may require additional support. The narrow confidence intervals demonstrate high precision in the population estimates.

**Table 2:** Prevalence of functional difficulties among children aged 2–17 years in Somalia [*n* = 25,078].

Functional difficulty	[*n* = 25,078]	Prevalence	95% CI
No functional difficulty	23,655	94.33%	[0.940, 0.946]
Yes, at least one functional difficulty	1,423	5.67%	[0.054, 0.060]

### Sociodemographic characteristics of the study population of children aged 2–17 years in Somalia

Table 3 presents sociodemographic characteristics of the study population. A total of 25,078 children were analysed in the study. The sex distribution of children was nearly equal, with 50.14% being female and 49.86% male. Regarding age, the largest proportion of children was aged 5–9 years, 35.26%, followed by those aged 10–14 years, 29.67%, 2–4 years, 21.33%, and 15–17 years, 13.75%. In terms of household composition, most children lived in large households with seven or more members 69.57%, while 28.71% resided in households with 4–6 members, and only 1.73% lived in small households of 1–3 members. Concerning health status, a small proportion of children, 3.03%, reported suffering from an illness in the month preceding the survey, while the vast majority, 96.97%, did not. Similarly, 0.90% of children were reported to have a chronic illness, whereas 99.10% had no chronic disease. With respect to education, just over half of the children, 50.45%, had ever attended school, while 49.55% had never been to school. Poverty status indicates that a substantial share of the sample lived in poor households 60.48%, compared to 39.52% residing in non-poor households. In terms of residence, 62.22% of children lived in urban areas, while 37.78% resided in rural areas. Regionally, children were fairly evenly distributed, with 38.78% residing in the Southern region, 37.94% in the Northern region, and 23.28% in the Central region.

**Table 3:** Sociodemographic characteristics of the study population of children aged 2–17 years in Somalia [*n* = 25,078].

Variable	Category	Weighted count	Weighted percent (%)
Sex of the child	Male	12,503	49.86
	Female	12,575	50.14
Age of the child	2–4 years	5,349	21.33
	5–9 years	8,842	35.26
	10–14 years	7,440	29.67
	15–17 years	3,447	13.75
Household size	1–3 members	433	1.73
	4–6 members	7,199	28.71
	≥7 members	17,446	69.57
Suffering illness	Yes	761	3.03
	No	24,317	96.97
School attendance	Yes	9,954	50.45
	No	9,775	49.55
Chronic illness status	Chronic disease	226	0.90
	No chronic disease	24,852	99.10
Poverty status	Not-poor	9,912	39.52
	Poor	15,166	60.48
Place of residence	Urban	15,604	62.22
	Rural	9,474	37.78
Region	Northern	9,514	37.94
	Central	5,839	23.28
	Southern	9,725	38.78

School attendance has missing values due to age limitations (children below school age).

### Bivariate associations between functional difficulty and selected sociodemographic characteristics among children aged 2–17 years in Somalia

Table 4 displays the bivariate association between disability status and selected socioeconomic and demographic characteristics among the study population (children aged 2–17 years) in Somalia. The selected predictors were sex, age, household size, suffering illness, school attendance, chronic diseases, poverty condition, as well as residence and region of the study population. Overall, disability was significantly associated with all determinant variables (*ρ* < 0.25). The most prevalent functional difficulties were in children aged 2–4 years, with 10.11% experiencing difficulties, while 89.89% had no such difficulties. Functional difficulty was slightly higher among males, 5.80%, compared to females, 5.55%. Children living in households with 1–3 members, 6.47% or 7 or more members, 5.77%, experienced slightly greater difficulties than those in households with 4–6 members, 5.40%. Among children with a reported illness, 10.91% had functional difficulties, compared with 5.51% among those without illness. Children not attending school had a higher prevalence of functional difficulty, 5.34%, than those attending school, 3.62%. Functional difficulty was markedly higher among children with chronic illnesses, 27.0%, compared to those without, 5.48%. Slightly higher difficulties were also observed among children from non-poor households, 6.39%, than in poor households, 5.21%. Rural children experienced more functional difficulty, 6.15%, than urban children, 5.38%. Regionally, children in the Central region had the lowest prevalence, 4.90%, while Northern and Southern regions had slightly higher rates, 5.94% and 5.88%, respectively.

**Table 4:** Bivariate associations between functional difficulty and selected sociodemographic characteristics among children aged 2–17 years in Somalia [*n* = 25,078].

Variable	Category	Functional difficulty				*ρ*-value
		Yes		No		
Sex of the child						<0.001
		%	[95% CI]	%	[95% CI]	
	Male	5.80	[0.054, 0.062]	94.20	[0.938, 0.946]	
	Female	5.55	[0.052, 0.060]	94.45	[0.940, 0.948]	
Age of the child						<0.001
	2–4 years	10.11	[0.093, 0.110]	89.89	[0.890, 0.907]	
	5–9 years	4.94	[0.045, 0.054]	95.06	[0.946, 0.955]	
	10–14 years	3.99	[0.036, 0.045]	96.01	[0.955, 0.964]	
	15–17 years	4.29	[0.037, 0.050]	95.71	[0.950, 0.963]	
Household size						<0.001
	1–3 members	6.47	[0.045, 0.092]	93.53	[0.908, 0.955]	
	4–6 members	5.40	[0.049, 0.060]	94.60	[0.940, 0.951]	
	≥7 members	5.77	[0.054, 0.061]	94.23	[0.939, 0.946]	
Suffering illness						<0.001
	Yes	10.91	[0.089, 0.133]	89.09	[0.867, 0.911]	
	No	5.51	[0.052, 0.058]	94.49	[0.942, 0.948]	
School attendance						<0.001
	Yes	3.62	[0.033, 0.040]	96.38	[0.960, 0.967]	
	No	5.34	[0.049, 0.058]	94.66	[0.942, 0.951]	
Chronic illness						<0.001
	Yes Chronic disease	26.99	[0.216, 0.332]	73.01	[0.668, 0.784]	
	No chronic disease	5.48	[0.052, 0.058]	94.52	[0.942, 0.948]	
Poverty status						<0.001
	Not-poor	6.39	[0.059, 0.069]	93.61	[0.931, 0.941]	
	Poor	5.21	[0.049, 0.056]	94.79	[0.944, 0.951]	
Residence						<0.001
	Urban	5.38	[0.050, 0.057]	94.62	[0.943, 0.950]	
	Rural	6.15	[0.057, 0.067]	93.85	[0.933, 0.943]	
Region						<0.001
	Northern	5.94	[0.055, 0.064]	94.06	[0.936, 0.945]	
	Central	4.90	[0.044, 0.055]	95.10	[0.945, 0.956]	
	Southern	5.88	[0.054, 0.064]	94.12	[0.936, 0.946]	

### Types of functional difficulties among children aged 2–17 years across six functional domains

**Figure 2:**
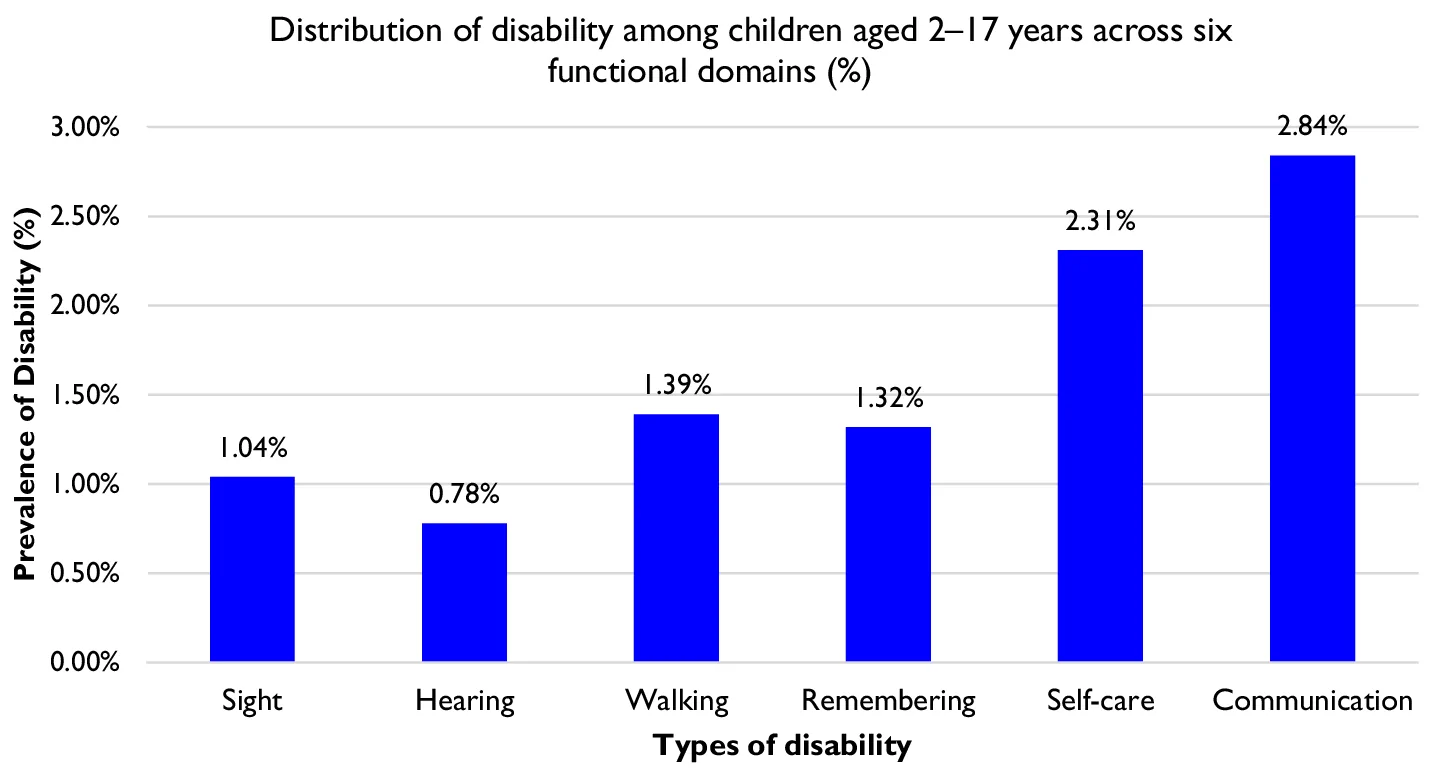
Types of functional difficulties among children aged 2–17 years across six functional domains [*n* = 25,078].

Figure 2 shows the types of disability among children aged 2–17 years across six functional domains (sight, hearing, walking, remembering, self-care, and communication). Communication/language-related functional difficulty was the most prevalent, affecting 2.84% of children, followed by difficulty in self-care at 2.31%. Disabilities related to walking, 1.39% and remembering, 1.32% were reported at moderate levels. Difficulty in sight or seeing affected 1.04% of children, while hearing-related difficulty was the least common, reported by 0.78% of the population. Overall, this indicates that communication and self-care challenges are more common among children than sensory or mobility-related disabilities.

### Multivariable logistic regression estimates of factors associated with functional difficulty (at least one functional difficulty) among Somali children aged 2–17 years

Table 5 demonstrates findings from a multiple logistic regression model. Several significant predictors of functional difficulty among children were identified. Household size, illness status, type of residence, school attendance, chronic illness status, and poverty status as statistically significant predictors of functional difficulty among children. Household size was significantly associated with functional difficulty. Children living in households with 4–6 members had 38% lower odds of functional difficulty compared to those living in households with 1–3 members (AOR = 0.622, 95% CI [0.435, 0.891], *ρ* = 0.010). Illness status was a strong determinant of functional difficulty. Children who were not suffering from illness had 54% lower odds of functional difficulty compared to those who were ill (AOR = 0.461, 95% CI [0.349, 0.608], *ρ* < 0.001). Similarly, the type of residence was also significantly associated with the outcome. Children residing in rural areas had 18% higher odds of functional difficulty compared to those living in urban areas (AOR = 1.180, 95% CI [1.017, 1.369], *ρ* = 0.029). School attendance showed a strong positive association with functional difficulty. Children who were not attending school had 59% higher odds of functional difficulty compared to those attending school (AOR = 1.586, 95% CI [1.363, 1.845], *ρ* < 0.001). Chronic illness status was the strongest predictor in the model. Children without a chronic disease had approximately 88% lower odds of functional difficulty compared to children with a chronic disease (AOR = 0.118, 95% CI [0.088, 0.159], *ρ* < 0.001). Finally, poverty status was significantly associated with functional difficulty. Children from poor households had 38% lower odds of functional difficulty compared to those from non-poor households (AOR = 0.620, 95% CI [0.533, 0.721], *ρ* < 0.001).

**Table 5:** Multivariable logistic regression estimates of factors associated with functional difficulty (at least one functional difficulty) among Somali children aged 2–17 years [*n* = 25,078].

Variable	Response	AOR	Std. Err.	95% CI	*ρ*-value
Sex of the child					
Reference	Male	1			
	Female	0.959	0.066	[0.837, 1.097]	0.540
Age of the child					
Reference	2–4 years	1			
	5–9 years	0.052	0.003	[0.047, 0.057]	0.102
	10–14 years	0.879	0.070	[0.751, 1.027]	0.105
	15–17 years	0.943	0.096	[0.773, 1.150]	0.564
Household size					
Reference	1–3 members	1			
	4–6 members	0.622	0.114	[0.435, 0.891]	**0.010**
	≥7 members	0.825	0.143	[0.587, 1.159]	0.268
Suffering illness					
Reference	Yes	1			
	No	0.461	0.065	[0.349, 0.608]	**0.001**
School attendance					
Reference	Yes	1			
	No	1.586	0.123	[1.363, 1.845]	**0.001**
Chronic illness					
Reference	Yes chronic disease	1			
	No chronic disease	0.118	0.018	[0.088, 0.159]	**0.001**
Poverty status					
Reference	Non-poor	1			
	Poor	0.620	0.048	[0.533, 0.721]	**0.001**
Type of residence					
Reference	Urban	1			
	Rural	1.180	0.090	[1.017, 1.369]	**0.029**
Region					
Reference	Northern	1			
Central	Central	1.195	0.114	[0.992, 1.441]	0.061
Southern	Southern	1.070	0.089	[0.909, 1.259]	0.416

Bolded variables indicate statistical significance at *p* < 0.05 in the model.

### Robustness analysis of the determinants of functional difficulty among Somali children aged 2–17 years

To validate the findings from the initial analysis, we employed a multiple probit model to assess the significant determinants of functional difficulty among children. The results, presented in Table 6, confirm that household size, suffering illness, school attendance status, residence type, chronic illness status, and poverty status are significant predictors of functional difficulty. Specifically, smaller household sizes, lack of school attendance, rural residence, presence of chronic illness, and poor household status were associated with higher odds of functional difficulty. These results align with previous findings, reinforcing the robustness of the identified predictors in explaining functional difficulty.

**Table 6:** Robustness check using a multivariable probit regression model for factors associated with functional difficulty among Somali children aged 2–17 years.

Variables	Coef.	SE	[95% CI]	*ρ*-Value
Sex of the child				
Male	1			
Female	−0.019	0.032	[−0.081, 0.043]	0.547
Age of the child				
1–4 years	1			
5–9 years	−0.375	0.032	[−0.439,-0.312]	0.102
10–14 years	−0.059	0.036	[−0.131, 0.012]	0.104
15–17 years	−0.038	0.046	[−0.129, 0.053]	0.410
Household size				
1–3 members	1			
4–6 members	−0.268	0.092	[−0.447,-0.088]	**0.001**
≥7 members	−0.142	0.088	[−0.315, 0.031]	0.108
Suffering illness				
Yes	1			
No	−0.398	0.071	[−0.538, −0.258]	**0.001**
School attendance				
Yes	1			
No	0.205	0.035	[0.135, 0.274]	**0.001**
Chronic illness				
Yes chronic disease	1			
No chronic disease	−1.166	0.084	[−1.331, −1.002]	**0.001**
Poverty status				
Non-poor	1			
Poor	−0.223	0.036	[−0.293, −0.154]	**0.001**
Residence				
Urban	1			
Rural	0.073	0.035	[0.006, 0.141]	**0.034**
Region				
Northern	1			
Central	0.083	0.044	[−0.003, 0.169]	0.058
Southern	0.026	0.038	[−0.048, 0.100]	0.486

Bolded variables indicate statistical significance at *p* < 0.05 in the model.

## Discussion

The findings of this study contribute to the growing evidence on childhood functional difficulties and their implications for child health and development in low-resource settings. The 2030 Sustainable Development Agenda recognizes disability inclusion as a central equity principle, emphasizing that children with disabilities and other vulnerable groups should not be left behind (29). However, the increasing burden of functional difficulties among children globally highlights the need for stronger identification, prevention, and support mechanisms (30). In this context, identifying populations at higher risk is essential for informing targeted interventions, improving access to health and educational services, and strengthening coordinated efforts among stakeholders (1, 31). By applying the Washington Group Child Functioning Module (CFM) framework (6), this study provides important evidence on the prevalence and determinants of functional difficulties among Somali children aged 2–17 years. These findings highlight the importance of integrating child functioning assessment into routine health and social services to promote early identification, appropriate support, and inclusive opportunities for children with functional difficulties.

### Prevalence and pattern of functional difficulties

The prevalence of functional difficulty among children aged 2–17 years in Somalia was 5.7% (95% CI: 5.4–6.0), indicating that these children experienced at least one functional limitation. Communication (2.84%) and self-care (2.31%) difficulties were the most common, while sight-related impairments were the least reported (0.78%). Although the overall prevalence appears relatively low, it still represents a substantial proportion of children requiring support. This estimate is comparable to findings from Bangladesh (5.5%) (30) and the World Report on Disability (32). However, it is higher than reports from northwest Ethiopia (5, 33), and lower than estimates from Cameroon (9.0%), Mexico (11.2%), India (12.0%), and South Asia (8.7%) (34) and India, 12.0% (1) and 8.7% in South Asia (35). These variations may be explained by differences in methodology, sample characteristics, age grouping, and contextual socioeconomic conditions. Improvements in healthcare access, early intervention, and socioeconomic conditions may also contribute to observed differences over time (33).

### Household and socioeconomic factors

Household size was significantly associated with functional difficulty. Children living in smaller households (1–3 members) had 38% lower odds of functional difficulty compared with those in households of 4–6 members (AOR = 0.622). This finding suggests that household structure may influence child functioning through variations in caregiving capacity, supervision, and resource availability. Smaller households may enable more individualized care, closer monitoring, and greater per-child resource allocation, whereas larger households may experience competing demands on caregivers’ time and household resources. Although evidence directly linking household size to child functional difficulty remains limited, previous studies have demonstrated that household socioeconomic conditions, caregiving environments, and family resources are important determinants of childhood disability and developmental outcomes. In sub-Saharan Africa, children with functional difficulties experience disparities in healthcare access and support services, partly influenced by household-level socioeconomic and caregiving factors (15, 16, 36). Furthermore, analyses of nationally representative surveys from low- and middle-income countries have shown that household wealth and living conditions are associated with child disability and functional limitations (37). These findings emphasize the importance of considering household characteristics in designing interventions to promote child functioning and disability-inclusive services (38). Poverty status was also a significant determinant. Children from non-poor households had 38% lower odds of functional difficulty compared to those from poor households (AOR = 0.620). Similar findings have been reported in previous studies (15, 39). With 54.4% of Somalia’s population living below the poverty line (40), socioeconomic deprivation may represent a critical structural determinant contributing to functional limitations among children by constraining access to essential healthcare services, adequate nutrition, and safe and supportive living environments.

### Health status and chronic conditions

Child health status showed a strong association with functional difficulty. Children who were not ill had 54% lower odds of functional difficulty compared to those who were ill (AOR = 0.460), consistent with prior evidence (16, 41). This finding is consistent with evidence from East Africa showing that childhood illness, chronic health conditions, and developmental problems increase the risk of functional limitations among children (42). Poor health status may impair physical, cognitive, and developmental functioning, emphasizing the importance of early diagnosis, treatment, and preventive healthcare interventions (38). Similarly, chronic illness emerged as the most influential determinant. Children without chronic conditions had approximately 88% lower odds of functional difficulty compared to those with chronic diseases (AOR = 0.120), consistent with previous studies (17, 43, 44). This highlights the long-term impact of chronic conditions on child functioning and the need for early diagnosis, treatment, and continuous care.

### School attendance and functional outcomes

School attendance was significantly associated with functional difficulty. Children not attending school had 59% higher odds of experiencing functional difficulties compared with those enrolled (AOR = 1.592). This finding is consistent with previous research demonstrating that children with disabilities or functional difficulties experience greater barriers to educational participation in low- and middle-income settings, including African contexts (15, 30, 42, 45, 46). This may reflect both the limiting effect of functional difficulties on school participation and the protective role of education in promoting cognitive, social, and physical development. In East Africa, evidence from Ethiopia and Uganda indicates that disability-related barriers, including limitations in functioning, inadequate educational support, and social exclusion, contribute to reduced school participation among children with disabilities. Conversely, access to education may provide protective benefits by strengthening cognitive development, social inclusion, and adaptive skills.

### Place of residence

Children residing in rural areas had higher odds of functional difficulty compared to their urban counterparts (AOR = 1.180), consistent with findings from Bangladesh (30, 47). This contrasts with evidence from Mexico, where disability prevalence was higher in urban areas (34). In Somalia, this rural disadvantage may reflect widespread poverty, limited access to healthcare, education, rehabilitation services, and essential infrastructure. With 65.5% of rural residents living below the poverty line (40), children in rural communities may face greater exposure to barriers such as long distances to services, shortages of trained providers, food insecurity, and inadequate developmental support, increasing their risk of functional difficulties.

## Strengths and limitations

This study uses nationally representative data to provide reliable estimates of disability prevalence among children aged 2–17 years in Somalia. Standardized and validated tools were applied to assess functional difficulties across six domains (vision, hearing, memory, mobility, self-care, and communication), contributing valuable evidence to the limited literature on childhood disability. Poverty status was used as a proxy for socioeconomic status because wealth indicators were missing. This study examines six functional domains from the 2022 SIHBS, which may not fully capture all aspects of child functioning and may therefore underestimate the burden of functional difficulties. The cross-sectional design limits causal inference, and reliance on self-reported data may introduce reporting bias. In addition, key factors such as maternal education and epilepsy were not included. Despite these limitations, the study provides important evidence on childhood functional difficulties in Somalia.

### Study implications

The study found that illness, school attendance, chronic conditions, household size, poverty, and residence type are key factors linked to functional difficulties among children in Somalia. Accordingly, the government should strengthen legal frameworks, policies, and awareness programs to improve the well-being of children with disabilities. Health authorities should expand community screening, mobile clinics, and early functional assessments, especially in underserved areas. The National Disability Agency and the Ministry of Health should implement targeted interventions to ensure access to healthcare, livelihoods, and protection services. The Ministry of Education should further advance inclusive education and literacy initiatives. Finally, social protection support should be scaled up for vulnerable households, and future studies should use multi-country and multi-survey data for deeper insights.

## Data Availability

The datasets presented in this study can be found in online repositories. The names of the repository/repositories and accession number(s) can be found here: https://microdata.nbs.gov.so/index.php/catalog/59/get-microdata.
